# From Pathophysiological Hypotheses to Case–Control Study Design: Resistance from Antibiotic Exposure in Community-Onset Infections

**DOI:** 10.3390/antibiotics11020201

**Published:** 2022-02-04

**Authors:** Salam Abbara, Didier Guillemot, Christian Brun-Buisson, Laurence Watier

**Affiliations:** 1 Anti-Infective Evasion and Pharmacoepidemiology Team, Inserm, UVSQ, University Paris-Saclay, CESP, 78180 Montigny-Le-Bretonneux, France; salam.abbara@gmail.com (S.A.); didier.guillemot@pasteur.fr (D.G.); chris.brunb@gmail.com (C.B.-B.); 2 Institut Pasteur, Epidemiology and Modelling of Antibiotic Evasion (EMAE), 75015 Paris, France; 3 Public Health, Medical Information, Clinical Research, AP-HP, University Paris Saclay, 94270 Le Kremlin-Bicêtre, France

**Keywords:** drug resistance, microbial, case–control studies, risk factors, anti-bacterial agents, public health

## Abstract

Antimicrobial resistance is a global public health concern, at least partly due to the misuse of antibiotics. The increasing prevalence of antibiotic-resistant infections in the community has shifted at-risk populations into the general population. Numerous case–control studies attempt to better understand the link between antibiotic use and antibiotic-resistant community-onset infections. We review the designs of such studies, focusing on community-onset bloodstream and urinary tract infections. We highlight their methodological heterogeneity in the key points related to the antibiotic exposure, the population and design. We show the impact of this heterogeneity on study results, through the example of extended-spectrum β-lactamases producing Enterobacteriaceae. Finally, we emphasize the need for the greater standardization of such studies and discuss how the definition of a pathophysiological hypothesis specific to the bacteria–resistance pair studied is an important prerequisite to clarify the design of future studies.

## 1. Introduction

Antimicrobial resistance (AMR) has emerged as a major public health threat with a high human and financial cost [1]. An abundant literature documents the link between antibiotic use, misuse, and the emergence of resistance (whether colonization or infection with resistant bacteria) [2]. Accordingly, appropriate antimicrobial use is at the core of all programs to fight AMR [1,3,4].

Over the last decades, resistance has widely spread in the community, shifting at-risk populations outside of hospitals. Salient examples are the community spread of extended-spectrum β-lactamases producing Enterobacteriaceae (ESBL-pE), or of USA300 methicillin-resistant *Staphylococcus aureus* (MRSA) [5,6]. Olesen et al. showed that population-wide resistance was more linked with broadly distributed antibiotic use, rather than with the intensity of use [7]. Thus, to better design control programs fighting AMR, it is important to clarify the link between antibiotic exposure and resistance in the community, where most antibiotic prescriptions occur [8]. This link should be evaluated depending on the bacteria–resistance pair. Indeed, for the same bacterial strain, various mechanisms interact at the individual and population level, leading to the emergence of a particular resistance in a given host [9]. Moreover, it is becoming increasingly clear that what is established for one bacterial species cannot be extrapolated to another, or even to different phylotypes within the same bacterial species [9].

Numerous epidemiological studies, mainly with a case–control design (comparing patients with resistant bacteria to patients with susceptible bacteria), have analyzed the link between antibiotic exposure and bacterial resistance, with various conclusions. Their methodological challenges—particularly the choice of the source population, control groups, and the measurement of antibiotic exposure—have already been discussed [10,11,12,13,14]. Definitions of colonization and infection are variable, and some studies combined them [15]. These limitations impact the interpretation of the study results and their integration into a global scheme specific to the bacteria–resistance pair of interest. Overall, it appears necessary to move towards the greater standardization of case–control studies looking at the link between antibiotic exposure and resistant infections. This standardization is even more important when studying the community context, as the study results may have an impact on the general population.

In this paper, we emphasize the key points and challenges involved in the construction of case–control studies studying the link between antibiotic exposure and resistant community-onset infections. We also discuss the importance of defining a pathophysiological hypothesis specific to the bacteria–resistance pair studied to clarify the design and interpretation of future studies. To this end, we review case–control studies evaluating the impact of antibiotic exposure on the occurrence of antibiotic-resistant community-onset (CO) urinary tract (UTI) or bloodstream (BSI) infections. In selected studies, we show the heterogeneity in the key points related to the risk factor of interest, the population and design: antibiotic exposure, cases, controls, and case–control design. We discuss the impact of this heterogeneity on the study results, focusing on ESBL-pE, often studied because of their rapid spread in the community, their propensity to colonize the human microbiota, and their morbidity and mortality [5]. Finally, we explain how to construct the pathophysiological hypothesis related to the bacteria–resistance pair studied, and how this translates when building a case–control study.

## 2. Studies’ Epidemiological Key Points: Challenges, Findings and Limitations

### 2.1. Risk Factor: Antibiotic Exposure

Four points need to be specified when studying antibiotic exposure [10]: 1. the choice and classification of antibiotics (by drug, class, or spectrum of activity); 2. the way antibiotic exposure was collected; 3. the selected window of exposure (period prior to patient inclusion, during which previous antibiotic exposure is assessed); 4. whether antibiotic exposure is considered with or without other criteria (quantification through defined daily doses, length of treatment, etc.).

#### 2.1.1. Classification

The classification of antibiotics was very heterogeneous among selected studies (Tables S1 and S2) [16,17,18,19,20,21,22,23,24,25,26,27,28,29,30,31,32,33,34,35,36,37,38,39,40,41,42,43,44,45,46,47,48,49,50,51,52,53,54,55,56,57,58,59,60,61,62]. Overall antibiotic exposure (i.e., without specifying a drug or class) was analyzed as a risk factor in 77% (36/47) of studies, while cephalosporins, (fluoro)quinolones, and penicillins were frequently individualized, with various sub-classifications. Note that studying antibiotics as a function of the drug, or globally, are complementary approaches: the first evaluates the link between (a) particular (class of) antibiotic(s) and the development of resistance for a bacteria–resistance pair; the second considers the overall impact of various antibiotic exposures on the process leading to resistance. Among all studies, 30% (14/47) individualized 5+ different (classes of) antibiotics. Moreover, 9% (4/47) individualized 10+ of them, without mentioning any method of adjustment to avoid alpha risk inflation due to multiple comparisons [31,41,52,58]. Anti-anaerobic antibiotics were individualized in 26% (12/47) of studies (“anti-anaerobic”, no. = 1; metronidazole, no. = 2; penicillins with β-lactamase inhibitors, no. = 9). Given the role of anaerobes as a barrier flora [63,64], isolating anti-anaerobic antibiotics as a distinct group in future studies should be informative.

#### 2.1.2. Collection of Antibiotic Exposure

The way in which antibiotic exposure was collected was not specified in 13% (6/47) of studies. More than half of the studies (27/47) assessed antibiotic exposure through medical records. This may induce a bias as the exposure to antibiotics is not systematically and homogeneously collected and traced in medical records. Patient interviews, which are subject to recall bias, were used in 13% (6/47) of studies. The assessment of delivered antibiotics in pharmacy databases was conducted in 9% (4/47) of studies. Because data reported by community pharmacies often relate to the dispensing of antibiotics, they may not reflect the actual consumption by patients.

#### 2.1.3. Window of Exposure

Among the 47 studies analyzed, 2% (1/47) did not define the window of exposure, and others used a window of exposure varying between 15 days and 1 year. Half of the studies (24/47) limited the window of exposure to 3 months. Only 21% of studies (10/47) considered windows of exposure longer than 3 months. Note that selecting the window of exposure is critical, particularly when addressing bacteria associated with persistent carriage. For instance, the duration of ESBL-pE carriage in the intestine may differ substantially according to the context of acquisition and population [65,66]. It could exceed 12 months in some subpopulations, such as critically ill patients or those exposed to antibiotics [66]. Thus, defining the window of exposure should be conducted according to the available information on the dynamics of the acquisition and loss of the studied resistant bacteria, and on the hypothetical source population. Another critical point when studying community-onset infections is to exclude antibiotics administered before inclusion for the patient’s current infection from prior antibiotic exposure. To this end, the window of exposure should exclude recent exposure to antibiotics. One study [33] explicitly mentioned excluding antibiotic exposure in the previous month from analysis. Defining the appropriate time limit between treatment of the infectious episode and prior antibiotic exposure can be difficult and depends on the studied infection and study setting. A sensitivity analysis testing several exposure windows could be considered, to test multiple hypotheses and add quantitative evidence to the influence of time from prescription to the occurrence of an antibiotic-resistant infection [57].

#### 2.1.4. Quantification of Antibiotic Exposure

Almost all reviewed studies evaluated prior exposure to antimicrobials (exposed vs. non-exposed), regardless of other criteria. One study defined antibiotic exposure as at least one standard dose in >24 h [37], a second defined it as the use of any antimicrobial agent for ≥3 days [28], and a third studied various doses and durations of treatment [57].

### 2.2. Populations and Design

#### 2.2.1. Cases: Community-Onset Infections

CO infections were commonly defined by their occurrence within the first 48 h of hospitalization (9/11 studies for CO-BSI, 3/9 studies for CO-UTI), sometimes excluding patients transferred from other hospitals or hospitalized within few weeks (Table S3) [67]. Other definitions considered outpatients, patients presenting at the emergency department (ED), or patients referred by their general practitioner. Another level of complexity is added when trying to define truly CA-infections, i.e., CO-infections that are not healthcare associated (HCA). While considering “CA-UTI”, 1/21 study did not define them. In other studies, the lability between CO and CA-infections was strong, as only 9/21 studies of CA-UTI excluded patients with exposure to healthcare.

The value of individualizing HCA-infections could be questioned. Its main clinical interest would be an increased risk of drug-resistant pathogens and mortality in HCA-infections, which may justify aggressive diagnostic and treatment measures [67]. However, taking the example of HCA-pneumonia (HCAP), the risk of AMR and adverse outcome attributable to HCAP appears to have been overestimated and may have resulted in over-treatment [68,69]. Yet, from an epidemiological point of view, individualizing HCA-infections seems useful. A study by Lin et al. identified antibiotic exposure as a risk factor for ESBL-pE BSI, but only in CO-BSI and not in CA-BSI [23]. Thus, repeating analyses done on patients with CO-infections in the subcategory of patients with CA-infections could better define the populations at risk.

The definitions of HCA-infections are diverse, and have been challenged for some infections, including pneumonia and BSI [67,69,70]. When prior exposure to healthcare was analyzed as a risk factor in the studies reviewed (11/11 in CO-BSI, 1/4 in CA-BSI, 17/21 in CA-UTI, 9/9 in CO-UTI), various definitions were considered: Friedman’s criteria [67], history of hospitalization or surgery or invasive urinary tract procedure (with various periods prior to admission), or residency in a nursing home or long-term care facility. If reaching a consensus on an operational definition of HCA infections seems difficult, case–control studies of CO-infections should at least clearly specify the definitions used for CO, CA and HCA-infections, and exclude patients with healthcare exposure from the CA group.

#### 2.2.2. Controls

In case–control studies, the definition of controls is a crucial step, depending on the specific question addressed. Most case–control designs define the control group as patients infected with a susceptible bacterium (to the studied antibiotic) [10,11]. This identifies the differential impact of antibiotic exposure on infection with the susceptible or resistant bacteria. For clinicians, such studies help determine the optimal empiric treatment for a patient with a presumed infection to the studied bacteria (e.g., to determine whether carbapenem treatment is warranted to cover ESBL-pE strains when selecting empiric therapy for UTI) [10,14].

To study community-acquired infections, it seems important to include a control group that approaches the uninfected source population. Such studies give valuable information on the impact of antibiotic exposure on the overall process leading individuals from the community to ultimately become infected. However, including uninfected patients can be very challenging, especially when it comes to identifying a representative sample within the community [10,11,12,13]. Moreover, the controls should have the same characteristics as those chosen by the authors to define community-acquired infections. Notably, the same criteria related to exposure to care (e.g., hospitalization in the previous week, recent surgery, or any other criteria) used for excluding cases should be applied to controls. Finally, depending on the studied pathogen and on the scientific question, this group could be the general population (if the pathogen is commensal), or uncolonized individuals from the general population for non-commensal species.

##### Patients Infected with Susceptible Bacteria

Two thirds of the retained studies addressed ESBL-pE CO or CA-UTI or BSI (29/47; BSI, no. = 8; UTI, no. = 21). Focusing on the 23 studies including a multivariate analysis (BSI, no. = 7, Table S4; UTI, no. = 16, Table S5), all included a control group having non-ESBL-pE infections [16,18,19,20,21,22,26,33,34,35,36,37,38,39,40,41,42,43,44,45,46,47,48]. Although they are usually considered risk factors for ESBL-pE, global exposure to antibiotics (median, min–max univariate odds ratio (OR): 6.3, 3.2–10.7 in BSI; 4.3, 2.0–10.1 in UTI), particularly cephalosporins (median, min–max OR: 6.07, 4.4–13.4 in BSI; 3.5, 1.4–17.1 in UTI) and quinolones (median, min–max OR: 6.4, 3.0–7.0 in BSI; 2.4, 0.9–5.8 in UTI), were risk factors in the multivariate analysis in only half of the studies that considered them (BSI, 3/4, 2/4, and 2/4 studies; UTI, 6/11, 5/10, and 4/10 studies, respectively). In UTI, exposure to other antibiotics, such as penicillin, macrolide or nitrofurantoin, were risk factors in a minority of studies (2/8, 1/2, 1/4, respectively). The exposure windows were variable, most being ≤3 months, precluding conclusions on the impact of antibiotics taken previously. In addition to differences in population characteristics, size, and setting, heterogeneity in the definition of exposure and cases may have contributed to these variable results. A small number of studies focused on CA-infections, with small numbers of patients and a wide methodological variability, which makes it impossible to assess if there are specificities in CA-infections compared to CO-infections.

##### Uninfected Hosts

No study with ESBL-pE BSI used uninfected controls. Only one study on ESBL-pE CO-UTI considered two control groups among which uninfected patients, randomly selected among residents from the same geographical are as cases [33].

#### 2.2.3. Designs

Most of the studies used a case–control design, except four studies that used a case–control(case)–control design. This design includes a third group of patients, to be considered as a second group of cases (if compared to the control group), or a second group of controls (if compared to the case group) [14]. In three studies, this third group included patients with sepsis [16], bacteremia of all causes [18], or infection with a bacterium that was different from the two other groups [35]. Only one study used a case–control–control design with both types of controls discussed previously, among which uninfected patients [33]. Such a design helps to identify whether antibiotic administration in individuals from the community selectively impacts the risk of developing a resistant infection, or the risk of having an infection, whether it is susceptible or resistant [14]. Indeed, comparing uninfected patients with both other groups allows to individualize the impact of antibiotic exposure on the occurrence of an infection to a resistant or a susceptible bacterium, independently. As such, the study by Søgaard et al. suggests that in an uninfected individual from the general population, prior exposure to broad-spectrum antibiotics, mecillinam, sulfamethizole and trimethoprim, could increase the risk of UTI to a non-ESBL or ESBL-pE. On the other hand, exposure to macrolides and nitrofurantoin could selectively increase the risk of ESBL-pE UTI [33].

## 3. Pathophysiological Hypotheses

To move towards greater standardization in future studies, we propose to construct and interpret them within a pathophysiological (i.e., causal) hypothesis specific to the bacteria–resistance pair of interest (Table 1). This hypothesis would detail the steps leading an uncolonized host from the community to present with an antibiotic-resistant infection. Authors could then embed their study within those steps. The hypothesis can be formulated at a macro-level (which can be embodied by different groups of patients, to help to design case–control studies), or a micro-level (considering intra-host and host-microbiota interactions and dynamics, adapted for other study designs). This strategy would help to design the study in such a way as to provide the most correct answer to the specific question addressed, while allowing the results to be interwoven with other study results. Ultimately, this would allow for a global view of the role of antibiotics at each step leading to an antibiotic-resistant infection, for a specific bacteria–resistance pair. It also allows the identification of transitions for which the impact of antibiotic exposure has not been studied, prompting additional studies.

### 3.1. Construction of the Pathophysiological Hypotheses

The pathophysiological hypothesis would detail the hypothetical causal chain leading an uninfected host to present an infection by the resistant bacteria. In order for an infection to occur in an individual, it is generally required that this individual has been previously colonized by this bacterium [64]. Whether the resistant bacteria can be acquired directly (as with MRSA), or whether it is generally necessary to go through a colonization stage by a susceptible strain secondarily acquiring resistance (as could be hypothesized for *Pseudomonas aeruginosa*), depends on the species and the resistance. Subsequently, infection is differentiated from colonization by the presence of clinical signs signaling the transition from asymptomatic carriage to infection [15].

Depending on the bacteria–resistance pair, a macro-level pathophysiological hypothesis could include four states, all of which could be embodied through specific patient groups: (1) no carriage of the pathogenic bacteria; (2) carriage of the susceptible pathogenic bacteria (e.g., colonization with a new phylotype of *Escherichia coli*, transient colonization by *Streptococcus pneumoniae* or *S. aureus*, transient or persistent colonization with *Enterobacter cloacae*, *Serratia* spp., *Klebsiella* spp.); (3) carriage of the resistant pathogenic bacteria (whether by mutation of a susceptible bacteria, acquisition from another host, or lateral gene transfer); (4) infection with the resistant pathogenic bacteria [9,15,71]. When designing a case–control study, clarifying the state(s) for which risk factors are being sought helps to define the most appropriate case and control groups (as proposed in Table 2).

### 3.2. An Example: ESBL-Producing E.coli ST131 CO-Infections

**Figure 1:**
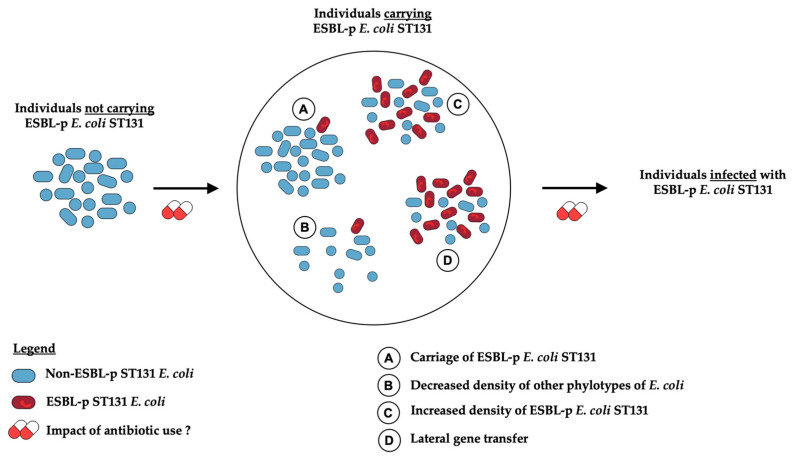
Pathophysiological hypothesis to study antibiotic exposure as a risk factor for infection with ESBL-producing *Escherichia coli* ST131.

To illustrate the interplay between the macro-level pathophysiological hypothesis and the design of case–control studies, we will focus on a specific bacteria–resistance pair, ESBL-producing (ESBL-p) *E. coli* sequence type 131 (ST131). ESBL-p *E. coli* ST131 is a pandemic multidrug resistant (MDR) strain that is expanding rapidly in the community [72]. To study risk factors for infection with ESBL-p *E. coli* ST131, a 4-step pathophysiological hypothesis beginning from the general population as the source population [72] could be proposed (Figure 1): (1) absence of ST131 *E. coli* carriage; (2) carriage of ST131 *E. coli* through individual cross-transmission, with transient colonization; (3) carriage of ESBL-p *E. coli* ST131 (after acquisition of ESBL gene-containing plasmids by horizontal transmission); (4) infection with ESBL-p *E. coli* ST131, for instance via intestinal translocation [72]. Understanding and weighing the potential role of antibiotics in the transition from one step to the next requires a specific design for each transition.

Morales Barroso et al. studied the role of antibiotics in the acquisition of ST131 *E. coli* (irrespective of ESBL production) in an uninfected host (step 1 to 2). They compared ST131 colonized and non-ST131 colonized household members of index community patients with *E. coli* ST131 infection and did not identify antibiotic exposure as a risk factor [73]. To our knowledge, no case–control study has used hosts carrying non-ESBL-p ST131 *E. coli* as controls, and ESBL-p *E. coli* ST131 as cases (step 2 to 3).

To evaluate the impact of antibiotic exposure in the transition from colonization to infection with ESBL-p *E. coli* ST131 (step 3 to 4), an optimal study design would include cases infected with ESBL-p *E. coli* ST131, and uninfected controls carrying ESBL-p *E. coli* ST131. To our knowledge, such a study has not been published. More generally, if antibiotic exposure has been shown to impact the risk of colonization to some MDR Enterobacteriaceae, its role in the progression from colonization to infection is partly understood [64]. Studies on this topic usually focus on hospitalized patients, especially the ICU setting, where colonization with MDR bacteria is often assessed [74]. To better address this question, it is important to design dedicated community-based studies with colonized individuals as controls and infected individuals as cases [75,76,77,78].

Some studies compared cases with ST131 and controls with non-ST131 ESBL-p *E. coli* infections [79,80]. This design specifically examines the impact of antibiotic exposure on the risk of developing an infection due to the ST131 clone, among patients who would develop ESBL-p *E. coli* infections.

It is notable that, while case–control studies evaluate risk factors involved in the transition from a step to another of the pathophysiological hypothesis, they do not discriminate the exact intra-host and host-microbiota mechanisms and dynamics involving the antibiotic in this transition, which are better approached through mathematical models and require microbiome analysis [10,81,82]. In the example discussed above, a micro-level pathophysiological hypothesis could involve further steps between asymptomatic carriage and infection with ESBL-p *E. coli* ST131 (Figure 1): (1) depletion in other phylotypes of *E. coli* in the host microbiota [64]; (2) increase in lateral gene transfer of the ESBL gene within or between bacterial species [81]; (3) increase in density of ESBL-p *E. coli* ST131 among the host’s flora [64] (which could be important for infections such as BSI [63]), with a loss of bacterial diversity. All these steps could be happening within the host microbiota of subjects carrying ESBL-p *E. coli* ST131 and be influenced by the selective pressure of antibiotics.

## 4. Materials and Methods

**Figure 2:**
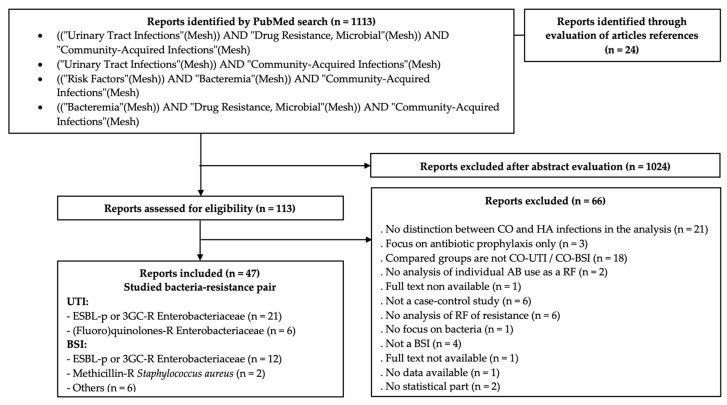
Flowchart of the selection process of case–control studies assessing the risk factors of resistance in bloodstream and urinary tract infections. Abbreviations: 3GC = third generation cephalosporins; AB = antibiotic; BSI = bloodstream infections; CO = community-onset; ESBL-p = Extended-Spectrum β-Lactamase producing; HA = hospital acquired; R = resistant; RF = risk factor; UTI = urinary tract infections.

We reviewed published case–control studies examining antibiotic exposure as a risk factor of resistance in CO-BSI (no. = 15) or UTI (no. = 32) (Table S1; Figure 2). MEDLINE was searched through PubMed until June 2020, with no language restriction or publication date limit, using MESH terms. The MESH term “Community-acquired infections” retrieved studies focusing on both community-acquired (CA) and community-onset (CO) infections. Titles and abstracts were screened to exclude those unrelated to the topic at hand. The remaining articles were assessed for eligibility. The reference lists of these articles were also searched for relevant titles. Only full-text articles were included. Full-text articles written in a language other than English or French were translated prior to analysis.

## 5. Conclusions

The increasing prevalence of antibiotic-resistant infections in the community, where most antibiotics are prescribed, makes it essential to clarify the link between antibiotic exposure in the community and the development of antibiotic-resistant bacterial infections. To this end, we emphasize the need to standardize future studies addressing this link. Indeed, those already published present a great heterogeneity in the definitions of antibiotic exposure, cases, and controls, impacting the interpretability of their results. Moreover, only a minority used uninfected controls, although these are essential to approach the source population of the infection and thus have a global view of the role of antibiotics on the occurrence of a resistant infection. In addition to the recommendations of previous methodological reviews, we encourage the authors of future studies to extend further and clearly define a pathophysiological hypothesis specific to the bacteria–resistance pair of interest. This preliminary step would help them to design their study, according to the specific question addressed. Even though an optimal design may be unrealistic, the pathophysiological hypotheses would provide a framework for discussing the pitfalls of the achieved design and comparing the study results with those of other studies.

## Figures and Tables

**Table 1:** Key points to design case–control studies evaluating antibiotic exposure as a risk factor of resistance in community-onset infections.

Identify the bacteria–resistance pair of interest. Define the pathophysiological hypothesis, clarifying the hypothetical uncolonized/uninfected source population and steps leading to infection. Determine the step(s) and the specific question that the study wants to address. Choose the control and case groups accordingly.

**Table 2:** Selecting the case and control groups according to the specific question.

Control Group	Case Group	Question Addressed
Uncolonized hosts	Hosts colonized with a given species	Assess the impact of antibiotic exposure on the risk of colonization.
Hosts colonized with the susceptible bacteria	Hosts colonized with the resistant bacteria	Assess the impact of antibiotic exposure on the apparition of a resistant strain within the colonizing susceptible strain.
Hosts colonized with a bacterial strain	Hosts infected with the same bacterial strain	Assess the impact of antibiotic exposure on the progression from colonization to infection.
Uninfected hosts	Hosts infected with the resistant bacteria	Assess the overall impact of antibiotic exposure on the process leading an uninfected host to present an infection to the studied resistant bacteria.
Control group 1: uninfected hosts Control group 2 ^@^: hosts infected with the susceptible bacteria	Hosts infected with the resistant bacteria	Assess whether antibiotic exposure selectively impacts the risk of developing a resistant infection, or the risk of having an infection, whether it is susceptible or resistant.
Hosts infected with the susceptible bacteria	Hosts infected with the resistant bacteria	Determine the most appropriate empiric treatment for a patient with a presumed infection to the studied bacteria. Assess the differential impact of antibiotic exposure on the processes leading a host to present with an antibiotic susceptible or resistant infection.

^@^ The control group 2 could be considered a case group 2, depending on the groups statistically compared by the study.

## Supplementary Materials

The following supporting information can be downloaded at: https://www.mdpi.com/article/10.3390/antibiotics11020201/s1. Table S1: Case–control studies assessing risk factors of resistance in community-onset or community-acquired bloodstream infections and urinary tract infections; Table S2: Classifications, windows of exposure and collections of antibiotic use in case–control studies assessing risk factors of resistance in community-onset or community-acquired bloodstream infections and urinary tract infections; Table S3: Definitions of community-onset and community-acquired infections in case–control studies assessing risk factors of resistance in bloodstream infections and urinary tract infections; Table S4: Main results of case–control studies assessing antibiotic use as a risk factor of resistance in CO-BSI or CA-BSI to ESBL-producing Enterobacteriaceae, as a function of the control group; Table S5: Main results of case–control studies assessing antibiotic use as a risk factor of resistance in CO-UTI or CA-UTI to ESBL-producing Enterobacteriaceae, as a function of the control group.
