# Supplementary material for: From Pathophysiological Hypotheses to Case–Control Study Design: Resistance from Antibiotic Exposure in Community-Onset Infections
Source: Antibiotics (Basel). 2022 Feb 4;11(2):201. doi: 10.3390/antibiotics11020201 (PMC8868523; doi:10.3390/antibiotics11020201)
Supplement: Supplementary file 1 [file antibiotics-11-00201-s001.zip › antibiotics-1566016-supplementary.pdf]

Table S1. Case-control studies assessing risk factors of resistance in community-onset or community-acquired bloodstream infections and urinary tract infections

First author, year	Studied group	Studied bacteria-resistance pair
Rodríguez-Baño, 2010 [1]	CO-BSI	ESBL-p *E. coli*
Hsieh, 2010 [2]	CO-BSI	ESBL-p *E. coli*
Park, 2011 [3]	CO-BSI	ESBL-p *E. coli*
Park, 2014 [4]	CO-BSI	ESBL-p *E. coli*
Kim, 2019 [5]	CO-BSI	ESBL-p *E. coli* or *Klebsiella* spp.
Lee, 2017 [6]	CO-BSI	ESBL-p Enterobacteriacea
Zahar, 2017 [7]	CO-BSI	ESBL-p Enterobacteriaceae
Lin, 2019 [8]	CO-BSI	3GC-R *E. coli*
Lee, 2014 [9]	CO-BSI	3GC-R *E. coli*, *K.pneumoniae*
Sung, 2012 [10]	CA-BSI (CO-like*)*	3GC-R *E. coli*, *Klebsiella, Enterobacter, Citrobacter*
Gottesman, 2018 [11]	CA-BSI	ESBL-p *E. coli*
Pedersen, 1999 [12]	CA-BSI	*E. Coli* R to ampicillin, sulphonamides, trimethoprim
Ortega, 2016 [13]	CA-BSI	3GC-R *E. coli* or *Klebsiella* spp.
Wi, 2018 [14]	CO-BSI	MRSA
Lim, 2014 [15]	CO-BSI	MRSA
Fan, 2014 [16]	CO-UTI	ESBL-p *E. coli*
Pérez Heras, 2017 [17]	CO-UTI	ESBL-p *E. coli*
Søgaard, 2017 [18]	CO-UTI	ESBL-p *E. coli*
Tüzün, 2019 [19]	CO-UTI	ESBL-p *E. coli*
Boix-Palop, 2017 [20]	CO-UTI	ESBL-p *K. pneumoniae*
Kung, 2015 [21]	CO-UTI	ESBL-p Enterobacteriacea
Calbo, 2006 [22]	CO-UTI (CA like)	ESBL-p *E. coli*
Zhu, 2019 [23]	CA-UTI (CO like)	ESBL-p *E. coli*
Topaloglu, 2010 [24]	CA-UTI (CO like)	ESBL-p *E. coli* or *Klebsiella* spp.
Yilmaz, 2008 [25]	CA/CO-UTI	ESBL-p *E. coli* or *K. pneumoniae*
Colodner, 2004 [26]	NH-UTI	ESBL-p *E. coli* or *Klebsiella* spp.
Albaramki, 2019 [27]	CA-UTI	ESBL-p *E. coli* or *Klebsiella* spp.
Guzmán, 2019 [28]	CA-UTI	ESBL-p *E. coli*
Koksal, 2019 [29]	CA-UTI	ESBL-p *E. coli* or *Klebsiella* spp.
Azap, 2010 [30]	CA-UTI	ESBL-p *E. coli*
Rodríguez-Baño, 2008 [31]	CA-UTI	ESBL-p *E. coli*
Castillo-Tokumori, 2017 [32]	CA-UTI	ESBL-p *E. coli*
Megged, 2014 [33]	CA-UTI	ESBL-p Enterobacteriacea
Chervet, 2018 [34]	CA-UTI (CO like)	ESBL-p Enterobacteriacea
Jacmel, 2017 [35]	CA-UTI (CO like)	ESBL-p Enterobacteriacea
Brosh-Nissimov, 2019 [36]	CA-UTI (CO like)	ESBL-p Enterobacteriacea
Colodner, 2008 [37]	CA-UTI (CO like)	*E. Coli* R to quinolones
Seija, 2014 [38]	CA-UTI (CO like)	*E. Coli* R to fluoroquinolones
Killgore, 2004 [39]	CA-UTI (CO like)	*E. Coli* R to ciprofloxacin
Chaniotaki, 2004 [40]	CA-UTI (CO like)	*E. Coli* R to quinolones
Dromigny, 2005 [41]	CA-UTI (CO like)	*E. Coli* R to ampicillin, amoxicillin, nalidixic acid, fluoroquinolones, TMP/SMX
Hillier, 2007 [42]	CA-UTI (CO like)	All bacteria, R to ampicillin, TMP
Anesi, 2016 [43]	CO-UTI	3GC-R Enterobacteriacea
Lee, 2015 [44]	CO-UTI	AmpC β-lactamase-producing Enterobacteriacea

Smithson, 2012 [45]	CA-UTI	*E. Coli* R to quinolones
Bosch-Nicolau, 2017 [46]	CA-UTI (CO like)	All bacteria, R to amoxicillin-clavulanate, cefuroxime, cefotaxime, ciprofloxacin
Filiatrault et al., 2012 [47]	CA-UTI	All bacteria, R to ciprofloxacin, TMP/SMX

***Table footnotes.** Abbreviations: 3GC = third generation cephalosporins; CA = community acquired; CO = community onset; CO-like = CA infection which definition actually corresponds to a CO infection; *E. coli = Escherichia coli;* ESBL-p = Extended-Spectrum β-Lactamase producing; *K. pneumoniae = Klebsiella pneumoniae;* MRSA = methicillin-resistant *Staphylococcus aureus;* NH = non-hospitalized; R = resistant; S = susceptible; SMX = sulfamethoxazole; TMP = trimethoprim.

Table S2. Classifications, windows of exposure and collections of antibiotic use in case-control studies assessing risk factors of resistance in community-onset or community-acquired bloodstream infections and urinary tract infections

	Bacteremia, no. (total=15)	UTI, no. (total = 32)
**Studied antibiotic(s) and sub classifications**		
Global use	13	23
Beta lactam antibiotics	3	2
Penicillins	4	12
Penicillins and beta-lactamase inhibitors	3	6
Cefuroxime / 2nd generation cephalosporins	3	5
Cephalosporins / 3rd generation cephalosporins	4	17
Narrow/broad-spectrum penicillins	0	1
Any of ampicillin, ampicillin/sulbactam, cefalotin	0	1
Carbapenems	1	3
Mecillinam	0	1
(Fluoro)quinolones	7	20
Cotrimoxazole	0	10
Nitrofurantoin	1	7
Aminosides	1	6
Macrolides	1	5
Clindamycin	0	1
Doxycyclin	0	1
Fosfomycin	0	3
Vancomycin	1	2
Anti-anaerobic	1	0
Metronidazole	0	2
Narrow/broad-spectrum antibiotics	0	1
**Window of exposure**		
15 days	1	0
1 month	4	5
2 months	1	0
3 months	7	17
6 months	1	4
12 months	1	3
"recent"	0	1
within 31-365 days before the index date	0	1
Not specified*	0	1
**Collection**		
Analysis of medical records	8	19
Patient interviews	0	6
Prospective evaluation without further details	3	1
Delivered antibiotics in pharmacy databases	2	2
Not specified*	2	4

***Table footnotes.**

* Some papers did not specify the window of exposure [40] or the way antibiotic exposure was collected [4,15,21,22,25,40].

Table S3. Definitions of community-onset and community-acquired infections in case-control studies assessing risk factors of resistance in bloodstream infections and urinary tract infections

**Studies focusing on "Community Onset" bacteremia (total = 11)**
<48h (no. = 4)
≤48h + not transferred from other hospitals (no. =3)
<48h + not hospitalized within 2 (no. =1) or 4 (no. =1) weeks before admission + not transferred from other hospitals
Residing in a long-term healthcare facility, or community-acquired (no. = 1)
Presenting at the ED (no. = 1)
**Studies focusing on "Community Acquired" bacteremia (total = 4)**
≤48h (no. = 1)
≤48h + not HCA (no. =2)
Present or incubating at admission to hospital + exclusion of patients hospitalized within the past 30 days (no. = 1)
**Studies focusing on "Community Onset" UTI (no. = 9)**
≤48h (no. = 3)
Presenting at the ED (no. = 1)
<48h or outpatients (no. = 1)
Presenting at the ED, or outpatient practices, or within 72 hours of hospital admission from the ED/outpatient clinic (no. = 1)
Presenting at the ED, and no hospitalization or long-term care facility stay within the preceding month (no. = 2)
Identified in a laboratory + referred by general practitioners (no. = 1)
**Study focusing on "Non hospitalized" UTI (no. = 1)**
**Study focusing on "Community onset/community acquired" UTI without further definition (no. = 1)**
**Studies focusing on "Community Acquired" UTI (no. = 21)**
No exclusion of HCA infections (no. = 11): <48h (no. = 1), presenting at the ED (no. = 3), GP practices (no. = 1), identified in an ambulatory laboratory (no. = 1), outpatients (no. = 5)
Exclusion of HCA infections (no. = 9): presenting at the ED or outpatients + not HCA (no. = 3), <48h + not HCA (no. = 1), <48h or ED + not HCA (no. =1), < = 72h + no hospitalization in the last two weeks prior to current UTI (no. = 1), not HCA (no. = 3)
Not specified (no. = 1) [30]

***Table footnotes.** Abbreviations: BSI = bloodstream infections; ED = emergency department; GP = general practitioner; HCA = healthcare associated; UTI = urinary tract infections.

Table S4. Main results of case-control studies assessing antibiotic use as a risk factor of resistance in CO-BSI or CA-BSI to ESBL-producing Enterobacteriacea, as a function of the control group

First author, year	Studied group	Number of cases	Number of controls	Exposure window	AB identified as a resistance RF	Odds ratio [95% CI]	Other antibiotics evaluated
Controls = non-ESBL-p Enterobacteriacea (no. = 10)							
Kim, 2019 [5]	CO-BSI	140	454	1 month	β-lactams or FQ	6·8 [2·3-20·2]	··
Lee, 2017 [6]	CO-BSI	65	1,076	1 month	Global use	15·3 [7·7–61·4]	··
Park, 2011* [3]	CO-BSI	50	100	3 months	Global use	4 [1·5-12·3]	··
Park, 2014† [4]	CO-BSI	60	180	3 months	3GC	16·4 [2·0-131·8]	1GC, 2GC, Penicillins, Quinolones
Rodríguez-Baño, 2010* [1]	CO-BSI	95	188	2 months	Global use<br>FQ<br>Cephalosporins	2·7 [1·5-4·9]<br>4·7 [2·0-11·1]<br>10·3 [2·1-50·3]	Aminopenicillins
Zahar, 2017 [7]	CA-BSI	22	335	1 year	··	··	β-Lactams, Cephalosporins, FQ, Carbapenems, Others, Macrolides, Nitrofurantoin, Aminoglycosides, Anti-anaerobic
Zahar, 2017 [7]	HCA-BSI	36	289	1 year	Anti-anaerobic	3·51 [1·6–7·5]	β-Lactams, Cephalosporins, FQ, Carbapenems, Others, Macrolides, Nitrofurantoin, Aminoglycosides,
Gottesman, 2018 [11]	CA-BSI	12	267	3 months	Quinolones	7 [1·7–29·4]	Global, Cefuroxime, Amoxicillin or penicillin, Amoxicillin/clavulanate
Controls = CO-sepsis (no. = 1)							
Rodríguez-Baño, 2010 [1]	CO-BSI	95	190	2 months	Global use<br>FQ	1·9 [1·03-3·5]<br>2·8 [1·2-6·5]	Aminopenicillins, Cephalosporins
Controls = CO-BSI (no. = 1)							
Park, 2011* [3]	CO-BSI	50	100	3 months	Global use	3·9 [1·3-11·5]	··

***Table footnotes.** A study without multivariate analysis was excluded [2].* = case-case(control)-control study, † = no information on how antibiotic use was evaluated. Abbreviations: 1GC, 2GC, 3GC = first, second, third generation cephalosporins; BSI = blood stream infections; CA = community acquired; CI = confidence interval; CO = community onset; CO-like = CA infection which definition actually corresponds to a CO infection; ESBL-p = Extended-Spectrum β-Lactamase producing; FQ = fluoroquinolones; RF = risk factor.

Table S5. Main results of case-control studies assessing antibiotic use as a risk factor of resistance in CO-UTI or CA-UTI to ESBL-producing Enterobacteriacea, as a function of the control group

First author, year	Studied group	Number of cases	Number of controls	Exposure window	AB identified as a resistance RF	Odds ratio [95% CI]	Other antibiotics evaluated
Controls = non-ESBL-p Enterobacteriacea (no. = 16)							
Søgaard, 2017* [18]	CO-UTI	339	3,390	Day -30 to -365	Macrolides<br>Nitrofurantoin	1·5 [1·1-2·2]<br>1·5 [1·1-2·3]	Mecillinam, Sulfamethizole, TMP, Narrow/ broad-spectrum penicillins, narrow/ broad-spectrum AB
Tüzün, 2019 [19]	CO-UTI	154	151	6 months	Global use	2·3 [1·2–4·3]	FQ, Cephalosporins, β-lactams + β-lactamase inhibitor
Zhu, 2019 [23]	CA-UTI (CO like)	111	103	3 months	Global use	4·2 [1·9–9·1]	··
Boix-Palop, 2017* [20]	CO-UTI	83	155	3 months	Cephalosporins<br>Quinolones	4·0 [1·8–9·2]<br>3·3 [1·4–7·9]	··
Kung, 2015† [21]	CO-UTI	62	376	3 months	··	··	Penicillins with/without β-lactamase inhibitor, Cephalosporins, FQ
Topaloglu, 2010 [24]	CA-UTI (CO like)	155	155	3 months	··	··	Global use, Penicillins, 2GC, 3GC+4GC, Others, Combinations, Suppression with TMP/SMX or nitrofurantoin
Yilmaz, 2008† [25]	CA/CO-UTI	62	62	3 months	Quinolones<br>Cephalosporins	··<br>··	Global use, Aminopenicillins, Aminoglycosides, Aminopenicillins + β-lactamase inhibitor, TMP/SMX
Colodner, 2004 [26]	NH-UTI	100	77	3 months	Global use<br>2GC<br>3GC<br>Quinolones<br>Penicillins	3·2 [1·8-5·9]<br>15·8 [1·7-143]<br>10·1 [4·2-24·0]<br>4·1 [1·8-9·0]<br>4·0 [1·6-9·0]	Macrolides, 1GC, TMP/SMX, Nitrofurantoin, Aminoglycosides
Calbo, 2006† [22]	CO-UTI (CA like)	19	55	1 year	Cefuroxime	21·4 [5·4–85·2]	3GC, Ciprofloxacin, Aminoglycosides

Albaramki, 2019 [27]	CA-UTI	77	77	3 months	··	··	Global use
Guzmán, 2019 [28]	CA-UTI	21	82	3 months	··	··	“First line β-lactams” (ampicillin, ampicillin/sulbactam, cefalotin)
Koksal, 2019 [29]	CA-UTI	66	112	3 months	··	··	Global use, Penicillins, Cephalosporins, FQ, Fosfomycin, Nitrofurantoin
Azap, 2010‡ [30]	CA-UTI	51	464	3 months	β-lactams	4·6 [2·0–10·7]	Global use, Quinolones
Rodríguez-Baño, 2008 [31]	CA-UTI	122 (93% had UTI)	244	3 months	Global use<br>Aminopenicillins<br>FQ<br>2GC + 3GC	2·9 [1·6-5·3]<br>2·8 [1·2-6·1]<br>1·9 [1·0-4·1]<br>3·2 [1·0-9·5]	Fosfomycin, Tromethamine
Castillo-Tokumori, 2017 [32]	CA-UTI	67	105	1 month	Global use	3·1 [1·4–6·7]	Penicillins, Cephalosporins, FQ
Megged, 2014 [33]	CA-UTI	80	80	1 month	Global use	4 [1·6–10·4]	··
Controls = uninfected patients from the general population (no. = 1)							
Søgaard, 2017* [18]	CO-UTI	339	3390	Day -30 to day -365	Macrolides<br>Nitrofurantoin<br>Broad-spectrum<br>AB<br>Mecillinam<br>Sulfamethizole<br>Trimethoprim	2·2 [1·3-3·8]<br>4·7 [1·9-11·2]<br>2·9 [1·1-7·7]<br>3·1 [1·3-7·8]<br>4·7 [2·4-9·2]<br>3·6 [1·6-8·0]	Narrow / broad-spectrum penicillins, narrow-spectrum AB

Table footnotes. Studies without multivariate analysis were excluded [16,17,34–36].
* = case-case(control)-control study, † = no information on how antibiotic use was evaluated, ‡ = no definition of CA infections.
Abbreviations: 1GC, 2GC, 3GC, 4GC = first, second, third, fourth generation cephalosporins; AB = antibiotics; CA = community acquired; CA-like = CO infection which definition actually corresponds to a CA infection; CI = confidence interval; CO = community onset; CO-like = CA infection which definition actually corresponds to a CO infection; ESBL-p = Extended-Spectrum β-Lactamase producing, FQ = fluoroquinolones; RF = risk factor; SMX = sulfamethoxazole; TMP = trimethoprim.

References

1. Rodríguez-Baño J, Picón E, Gijón P, Hernández JR, Ruíz M, Peña C, Almela M, et al. Community-Onset Bacteremia Due to Extended-Spectrum β-Lactamase–Producing Escherichia coli: Risk Factors and Prognosis. CLIN INFECT DIS. 2010 Jan;50(1):40–8.

2. Hsieh C-J, Shen Y-H, Hwang K-P. Clinical Implications, Risk Factors and Mortality Following Community-onset Bacteremia Caused by Extended-spectrum β-lactamase (ESBL) and non-ESBL Producing Escherichia coli. Journal of Microbiology, Immunology and Infection. 2010 Jun;43(3):240–8.

3. Park SH, Choi S-M, Lee D-G, Kim J, Choi J-H, Kim S-H, et al. Emergence of Extended-Spectrum β-Lactamase-Producing Escherichia coli as a Cause of Community-Onset Bacteremia in South Korea: Risk Factors and Clinical Outcomes. Microbial Drug Resistance. 2011 Dec;17(4):537–44.

4. Park YS, Bae IK, Kim J, Jeong SH, Hwang S, Seo Y-H, et al. Risk Factors and Molecular Epidemiology of Community-Onset Extended-Spectrum β-Lactamase-Producing Escherichia coli Bacteremia. Yonsei Med J. 2014;55(2):467.

5. Kim M, Song K-H, Kim C-J, Choe PG, Park WB, Bang JH, et al. Clinical Prediction Score for Community-Onset Bloodstream Infections Caused by Extended-Spectrum Beta-Lactamase-Producing *Escherichia coli* and *Klebsiella* Species. J Korean Med Sci. 2019;34(14):e116.

6. Lee C-H, Chu F-Y, Hsieh C-C, Hong M-Y, Chi C-H, Ko W-C, et al. A simple scoring algorithm predicting extended-spectrum β-lactamase producers in adults with community-onset monomicrobial Enterobacteriaceae bacteremia: Matters of frequent emergency department users. Medicine. 2017 Apr;96(16):e6648.

7. Zahar J-R, Lesprit P, Ruckly S, Eden A, Hikombo H, Bernard L, et al. Predominance of healthcare-associated cases among episodes of community-onset bacteraemia due to extended-spectrum β-lactamase-producing Enterobacteriaceae. International Journal of Antimicrobial Agents. 2017 Jan;49(1):67–73.

8. Lin W-P. Prevalence of and risk factor for community-onset third-generation cephalosporin-resistant Escherichia coli bacteremia at a medical center in Taiwan. 2019;11.

9. Lee S, Han SW, Kim KW, Song DY, Kwon KT. Third-generation cephalosporin resistance of community-onset Escherichia coli and Klebsiella pneumoniae bacteremia in a secondary hospital. Korean J Intern Med. 2014;29(1):49.

10. Sung YK, Lee JK, Lee KH, Lee KT, Kang C-I. The Clinical Epidemiology and Outcomes of Bacteremic Biliary Tract Infections Caused by Antimicrobial-Resistant Pathogens: American Journal of Gastroenterology. 2012 Mar;107(3):473–83.

11. Gottesman B-S, Shitrit P, Katzir M, Chowers M. Antibiotic Exposure in the Community and Resistance Patterns of Escherichia coli Community-Acquired Bloodstream Infection. Isr Med Assoc J. 2018 Jun;20(6):382–4.

12. Pedersen G, Schønheyder HC, Steffensen FH, Sørenson HT. Risk of resistance related to antibiotic use before admission in patients with community-acquired bacteraemia. Journal of Antimicrobial Chemotherapy. 1999 Jan;43(1):119–26.

13. Ortega M, Soriano A, Marco F, Almela M, Martínez JA, Morata L, et al. Risk factors for the isolation of a third generation cephalosporin resistant strain in patients with community-acquired Enterobacteriaceae bacteraemia. J Infect. 2016 Feb;72(2):268–71.

14. Wi YM, Rhee JY, Kang CI, Chung DR, Song JH, Peck KR. Clinical predictors of methicillin-resistance and their impact on mortality associated with Staphylococcus aureus bacteraemia. Epidemiol Infect. 2018 Jul;146(10):1326–36.

15. Lim CJ, Cheng AC, Kong DC, Peleg AY. Community-onset bloodstream infection with multidrug-resistant organisms: a matched case-control study. 2014;9.

16. Fan N-C, Chen H-H, Chen C-L, Ou L-S, Lin T-Y, Tsai M-H, et al. Rise of community-onset urinary tract infection caused by extended-spectrum β-lactamase-producing Escherichia coli in children. Journal of Microbiology, Immunology and Infection. 2014 Oct;47(5):399–405.

17. Pérez Heras I, Sanchez-Gomez JC, Beneyto-Martin P, Ruano-de-Pablo L, Losada-Pinedo B. Community-onset extended-spectrum β-lactamase producing Escherichia coli in urinary tract

infections in children from 2015 to 2016: Prevalence, risk factors, and resistances. Medicine (Baltimore). 2017 Dec;96(50):e8571.
18. Søgaard M, Heide-Jørgensen U, Vandenbroucke JP, Schønheyder HC. Risk factors for extended-spectrum b-lactamase-producing Escherichia coli urinary tract infection in the community in Denmark: a caseecontrol study. Clinical Microbiology and Infection. 2017;9.
19. Tüzün T, Sayın Kutlu S, Kutlu M, Kaleli İ. Risk factors for community-onset urinary tract infections caused by extended-spectrum β-lactamase-producing Escherichia coli. Turk J Med Sci. 2019 Aug 8;49(4):1206–11.
20. Boix-Palop L, Xercavins M, Badía C, Obradors M, Riera M, Freixas N, et al. Emerging extended-spectrum β-lactamase-producing Klebsiella pneumoniae causing community-onset urinary tract infections: a case–control–control study. International Journal of Antimicrobial Agents. 2017 Aug;50(2):197–202.
21. Kung C-H, Ku W-W, Lee C-H, Fung C-P, Kuo S-C, Chen T-L, et al. Epidemiology and risk factors of community-onset urinary tract infection caused by extended-spectrum β-lactamase-producing Enterobacteriaceae in a medical center in Taiwan: A prospective cohort study. Journal of Microbiology, Immunology and Infection. 2015 Apr;48(2):168–74.
22. Calbo E, Romaní V, Xercavins M, Gómez L, Vidal CG, Quintana S, et al. Risk factors for community-onset urinary tract infections due to Escherichia coli harbouring extended-spectrum β-lactamases. Journal of Antimicrobial Chemotherapy. 2006 Apr 1;57(4):780–3.
23. Zhu FH, Rodado MP, Asmar BI, Salimnia H, Thomas R, Abdel-Haq N. Risk factors for community acquired urinary tract infections caused by extended spectrum β-lactamase (ESBL) producing Escherichia coli in children: a case control study. Infectious Diseases. 2019 Dec 2;51(11–12):802–9.
24. Topaloglu R, Er I, Dogan BG, Bilginer Y, Ozaltin F, Besbas N, et al. Risk factors in community-acquired urinary tract infections caused by ESBL-producing bacteria in children. Pediatr Nephrol. 2010 May;25(5):919–25.
25. Yilmaz E, Akalin H, Özbey S, Kordan Y, Sinirtaş M, Gürcüoglu E, et al. Risk Factors in Community-Acquired/Onset Urinary Tract Infections Due to Extended-Spectrum Beta-Lactamase-Producing Escherichia coli and Klebsiella pneumoniae. Journal of Chemotherapy. 2008 Oct;20(5):581–5.
26. Colodner R, Rock W, Chazan B, Keller N, Guy N, Sakran W, et al. Risk Factors for the Development of Extended-Spectrum Beta-Lactamase-Producing Bacteria in Nonhospitalized Patients. European Journal of Clinical Microbiology & Infectious Diseases. 2004 Mar 1;23(3):163–7.
27. Albaramki JH, Abdelghani T, Dalaeen A, Khdair Ahmad F, Alassaf A, Odeh R, et al. Urinary tract infection caused by extended-spectrum β-lactamase-producing bacteria: Risk factors and antibiotic resistance. Pediatrics International. 2019 Nov;61(11):1127–32.
28. Guzmán M, Salazar E, Cordero V, Castro A, Villanueva A, Rodulfo H, et al. Multidrug resistance and risk factors associated with community-acquired urinary tract infections caused by Escherichia coli in Venezuela. biomedica. 2019 May 1;39:96–107.
29. Koksal E, Tulek N, Sonmezer MC, Temocin F, Bulut C, Hatipoglu C, et al. Investigation of risk factors for community-acquired urinary tract infections caused by extended-spectrum beta-lactamase *Escherichia coli* and *Klebsiella* species. Investig Clin Urol. 2019;60(1):46.
30. Azap ÖK, Arslan H, Şerefhanoğlu K, Çolakoğlu Ş, Erdoğan H, Timurkaynak F, et al. Risk factors for extended-spectrum β-lactamase positivity in uropathogenic Escherichia coli isolated from community-acquired urinary tract infections. Clinical Microbiology and Infection. 2010 Feb;16(2):147–51.
31. Rodríguez-Baño J, Alcalá JC, Cisneros JM, Grill F, Oliver A, Horcajada JP, et al. Community Infections Caused by Extended-Spectrum β-Lactamase–Producing Escherichia coli. Arch Intern Med. 2008 Sep 22;168(17):1897.
32. Castillo-Tokumori F, Irey-Salgado C, Málaga G. Worrisome high frequency of extended-spectrum beta-lactamase-producing Escherichia coli in community-acquired urinary tract infections: a

case–control study. International Journal of Infectious Diseases. 2017 Feb;55:16–9.
33. Megged O. Extended-spectrum β-lactamase-producing bacteria causing community-acquired urinary tract infections in children. Pediatr Nephrol. 2014 Sep;29(9):1583–7.
34. Chervet D, Lortholary O, Zahar J-R, Dufougeray A, Pilmis B, Partouche H. Antimicrobial resistance in community-acquired urinary tract infections in Paris in 2015. Médecine et Maladies Infectieuses. 2018 May;48(3):188–92.
35. Jacmel L, Timsit S, Ferroni A, Auregan C, Angoulvant F, Chéron G. Extended-spectrum β-lactamase-producing bacteria caused less than 5% of urinary tract infections in a paediatric emergency centre. Acta Paediatr. 2017 Jan;106(1):142–7.
36. Brosh-Nissimov T, Navon-Venezia S, Keller N, Amit S. Risk analysis of antimicrobial resistance in outpatient urinary tract infections of young healthy adults. Journal of Antimicrobial Chemotherapy. 2019 Feb 1;74(2):499–502.
37. Colodner R, Kometiani I, Chazan B, Raz R. Risk Factors for Community-Acquired Urinary Tract Infection Due to Quinolone-Resistant E. coli. Infection. 2008 Feb;36(1):41–5.
38. Seija V, Fratchez V, Ventura V, Pintos M, González M. Risk factors for community-acquired urinary tract infection caused by fluoroquinolone resistant E. coli. Rev Chilena Infectol. 2014 Aug;31(4):400–5.
39. Killgore KM, March KL, Guglielmo BJ. Risk Factors for Community-Acquired Ciprofloxacin-Resistant Escherichia Coli Urinary Tract Infection. Ann Pharmacother. 2004 Jul;38(7–8):1148–52.
40. Chaniotaki S, Giakouppi P, Tzouvelekis LS, Panagiotakos D, Kozanitou M, Petrikkos G, et al. Quinolone resistance among Escherichia coli strains from community-acquired urinary tract infections in Greece. Clinical Microbiology and Infection. 2004 Jan;10(1):75–8.
41. Dromigny JA, Nabeth P, Juergens-Behr A, Perrier-Gros-Claude JD. Risk factors for antibiotic-resistant Escherichia coli isolated from community-acquired urinary tract infections in Dakar, Senegal. Journal of Antimicrobial Chemotherapy. 2005 Jul 1;56(1):236–9.
42. Hillier S, Roberts Z, Dunstan F, Butler C, Howard A, Palmer S. Prior antibiotics and risk of antibiotic-resistant community-acquired urinary tract infection: a case–control study. Journal of Antimicrobial Chemotherapy. 2007 Jul 1;60(1):92–9.
43. Anesi JA, Lautenbach E, Nachamkin I, Garrigan C, Bilker WB, Wheeler M, et al. Clinical and Molecular Characterization of Community-Onset Urinary Tract Infections Due to Extended-Spectrum Cephalosporin-Resistant Enterobacteriaceae. Infect Control Hosp Epidemiol. 2016 Dec;37(12):1433–9.
44. Lee C-H, Lee Y-T, Kung C-H, Ku W-W, Kuo S-C, Chen T-L, et al. Risk factors of community-onset urinary tract infections caused by plasmid-mediated AmpC β-lactamase-producing Enterobacteriaceae. Journal of Microbiology, Immunology and Infection. 2015 Jun;48(3):269–75.
45. Smithson A, Chico C, Ramos J, Netto C, Sanchez M, Ruiz J, et al. Prevalence and risk factors for quinolone resistance among Escherichia coli strains isolated from males with community febrile urinary tract infection. Eur J Clin Microbiol Infect Dis. 2012 Apr;31(4):423–30.
46. Bosch-Nicolau P, Falcó V, Viñado B, Andreu A, Len O, Almirante B, et al. A Cohort Study of Risk Factors That Influence Empirical Treatment of Patients with Acute Pyelonephritis. Antimicrob Agents Chemother. 2017 Dec;61(12):e01317-17, e01317-17.
47. Filiatrault L, McKay RM, Patrick DM, Roscoe DL, Quan G, Brubacher J, et al. Antibiotic resistance in isolates recovered from women with community-acquired urinary tract infections presenting to a tertiary care emergency department. CJEM. 2012 Sep;14(5):295–305.
